# Circular RNA circ_0128846 promotes the progression of osteoarthritis by regulating miR-127-5p/NAMPT axis

**DOI:** 10.1186/s13018-021-02428-z

**Published:** 2021-05-11

**Authors:** Chao Liu, Ping Cheng, Jianjun Liang, Xiaoming Zhao, Wei Du

**Affiliations:** 1 Department of Emergency, 3201 Hospital, Hanzhong, Shaanxi China; 2 Department of Emergency, Binzhou People’s Hospital, No. 515 Huanghe Seven Road, Bincheng District, Binzhou City, 256610 Shandong Province China; 3 Department of Orthopaedics, Baoji Central Hospital, No. 8 Jiangtan Road, Weibin District, Baoji, 721008 Shaanxi Province China

**Keywords:** Osteoarthritis, circ_0128846, miR-127-5p, NAMPT

## Abstract

**Background:**

Mounting evidence indicates that circular RNAs (circRNAs) participate in the occurrence and development of various diseases, including osteoarthritis (OA). However, the effects and molecular mechanism of circ_0128846 in OA have not been reported.

**Methods:**

The expression levels of circ_0128846, microRNA-127-5p (miR-127-5p), and nicotinamide phosphoribosyltransferase (NAMPT) were determined by quantitative real-time polymerase chain reaction (qRT-PCR) or western blot assay. Cell viability was determined by Cell Counting Kit-8 (CCK-8) assay. Cell apoptosis was examined by flow cytometry and western blot assay. Inflammatory response and cartilage extracellular matrix (ECM) degradation were evaluated by western blot assay. The relationship between miR-127-5p and circ_0128846 or NAMPT was predicted by bioinformatics tools and verified by dual-luciferase reporter and RNA Immunoprecipitation (RIP) assays.

**Results:**

Circ_0128846 and NAMPT were upregulated and miR-127-5p was downregulated in OA cartilage tissues. Knockdown of circ_0128846 increased cell viability and inhibited apoptosis, inflammation and ECM degradation in OA chondrocytes, while these effects were reversed by downregulating miR-127-5p. Moreover, circ_0128846 positively regulated NAMPT expression by sponging miR-127-5p. Furthermore, miR-127-5p promoted cell viability and suppressed apoptosis, inflammation, and ECM degradation in OA chondrocytes by directly targeting NAMPT.

**Conclusion:**

Circ_0128846 knockdown might inhibit the progression of OA by upregulating miR-127-5p and downregulating NAMPT, offering a new insight into the potential application of circ_0128846 in OA treatment.

**Supplementary Information:**

The online version contains supplementary material available at 10.1186/s13018-021-02428-z.

## Highlights

Circ_0128846 was upregulated in OA cartilage tissues.

Circ_0128846 knockdown inhibited the progression of OA by upregulating miR-127-5p.

MiR-127-5p overexpression suppressed the progression of OA by targeting NAMPT.

Circ_0128846 regulated NAMPT expression in OA chondrocytes by sponging miR-127-5p.

## Introduction

Osteoarthritis (OA) is one of the most common joint diseases, and it is the leading cause of mobility-associated disability [1]. The main characteristics of OA are degradation of the articular cartilage due to the degeneration of cartilage extracellular matrix (ECM), as well as subchondral bone sclerosis and osteophyte formation [2]. Although great progress has been made, there is no effective treatment for OA [3]. OA progression is usually associated with inflammatory responses, and the major pro-inflammatory and pro-catabolic cytokines can induce matrix metalloproteinase (MMP) release [4]. Thus, it is essential to study the complex pathogenesis of OA for better prevention and treatment of OA.

Circular RNAs (circRNAs) are a special type of non-coding RNAs, which has received extensive attention in recent years [5]. CircRNA forms a continuous covalently closed loop without 5′-end cup and 3′-end ploy A tail (unlike lncRNA) [6]. Recently, more and more researchers have found that circRNAs are involved in modulation of gene expression and the development and progression of multiple diseases, including OA [7, 8]. For example, circSERPINE2, circGCN1L1 and circPSMC played pivotal roles in regulating OA chondrocyte growth, differentiation and apoptosis [9–11] CircRNA circ_0128846 (chr5:32379220-32420208) is derived from back-splicing of zinc finger RNA binding (ZFR) transcript and has been suggested to be upregulated in OA [12]. However, the exact roles and regulatory mechanism of circ_0128846 in OA have not been reported.

It is generally believed that circRNAs are enriched in microRNA (miRNA)-binding sites and can serve as competing endogenous RNAs (ceRNAs) or miRNA sponges to inhibit miRNA activity by competitively binding to miRNAs [13]. MiRNAs usually bind to the 3′untranslated regions (3′UTR) of target mRNAs to inhibit target gene expression [14]. It has been reported that dysregulation of miRNAs is strongly related to multiple pathological processes, including OA [15–19]. MiR-127-5p has been shown to be downregulated in OA and act as a key modulator of the MMP13 and catabolic signaling pathways in human chondrocytes [20]. Moreover, nicotinamide phosphoribosyltransferase (NAMPT; also known as visfatin) is an essential catabolic regulator of OA and participates in inflammatory pathways of OA [21, 22]. However, there is no report on the relationships among circ_0128846, miR-127-5p and NAMPT in the progression of OA.

In this research, circ_0128846, miR-127-5p, and NAMPT abundance were measured in OA cartilage tissues. Moreover, we explored the effects of circ_0128846, miR-127-5p, and NAMPT on cell viability, apoptosis, inflammation, and ECM degradation and determined their relationships in OA chondrocytes. Collectively, our research focused on uncovering the role of circ_0128846/miR-127-5p/NAMPT axis in OA chondrocytes.

## Materials and methods

### Specimens collection

OA cartilage tissues (*n* = 21) were collected from OA patients who underwent total knee arthroplasty at 3201 Hospital, as well as normal cartilage tissues (*n* = 8) were obtained from amputated patients with no history of OA or rheumatoid arthritis. These subjects have signed the informed consents. After surgical resection, these tissues were timely frozen in liquid nitrogen and subsequently preserved at − 80 °C until usage. This research had been granted by the Research Ethics Committee of 3201 Hospital.

### OA chondrocyte isolation and culture

OA chondrocytes were separated from OA cartilage tissues as previously described [23, 24]. In short, cartilage tissues were cut into small chippings (~ 0.5 cm^2^), and digested with trypsin (0.25%, Gibco, Grand Island, NY, USA) at 37 °C for 1 h, followed by incubation with collagenase Type II (0.2%, Millipore, Billerica, MA, USA) at 37°C for 3-4 h. After centrifugation, primary cells were collected and maintained in Dulbecco’s modified Eagle’s medium (DMEM, Gibco) containing 10% fetal bovine serum (FBS; Invitrogen, Carlsbad, CA, USA) in incubator at 37 °C with 5% CO_2_.

### Transfection

The small interfering RNAs (siRNAs) against circ_0128846 (si-circ_0128846#1, si-circ_0128846#2, and circ_0128846#3) and corresponding control (si-NC), miR-127-5p mimic (miR-127-5p) and corresponding control (NC), miR-127-5p inhibitor (anti-miR-127-5p) and corresponding control (anti-NC), NAMPT overexpression vector (NAMPT) and corresponding control (vector) were obtained from Genecreat (Wuhan, China). OA chondrocyte transfection was performed using the Lipofectamine 3000 reagent (Invitrogen).

### Quantitative real-time polymerase chain reaction (qRT-PCR)

Total RNA was isolated from OA cartilage tissues, normal tissues, and OA chondrocytes using the TRIzol reagent (Invitrogen). Next, complementary DNA (cDNA) was synthesized by One Step PrimeScript cDNA Synthesis Kit (Takara, Tokyo, Japan) and TaqMan microRNA Reverse Transcription Kit (Applied Biosystems, Foster City, CA, USA), respectively. Then, qRT-PCR reactions were performed on ABI Prism 7900HT (Applied Biosystems) using SYBR-Green Real-Time PCR Kit (Takara). In this study, primers used for amplification were listed as follows: circ_0128846, 5′-GACCTCTGTCAGCGAGTTCC-3′ (F) and 5′-AGCTACTGGAGCCTGATGGA-3′ (R); ZFR, 5′-TCCCAATGCTAAGGAGATGC-3′ (F) and 5′-TTCTTCTCGTCTTCGCCAGT-3’ (R); miR-127-5p, 5′-GCCGAGCTGAAGCTCAGAGG-3’ (F) and 5′-CTCAACTGGTGTCGTGGA-3’ (R); NAMPT, 5′-ATCCTGTTCCAGGCTATTCTG-3’ (F) and 5′-CCCCATATTTTCTCACACGCAT-3’ (R); glyceraldehyde-3-phosphate dehydrogenase (GAPDH), 5′-GTCTCCTCTGACTTCAACAGCG-3’; (F) and 5′-ACCACCCTGTTGCTGTAGCCAA-3′ (R); U6, 5′-CTCGCTTCGGCAGCACATATACT-3′ (F) and 5′-ACGCTTCACGAATTTGCGTGTC-3′ (R). The expression of genes was evaluated with 2^−ΔΔCt^ method. GAPDH and U6 were served as the internal references.

### RNase R treatment

To remove linear RNA, total RNA (5 μg) was incubated using 3 units of RNase R (Epicentre Biotechnologies, Madison, WI, USA) for 0.5 h at 37 °C. Afterward, the RNA expression of circ_0128846 and liner mRNA (ZFR) was detected using qRT-PCR analysis.

### Cell viability assay

Cell counting Kit-8 (CCK-8; Beyotime, Jiangsu, China) was employed for measuring cell viability. In short, OA chondrocytes (4 × 10^4^ cells/well) were placed in 96-well plates. CCK-8 (10 μL) was added to each well at pointed times. After incubation for 2–3 h, the absorbance at 450 nm wavelength was utilized to assess cell viability.

### Flow cytometry

Annexin V-fluorescein isothiocyanate (FITC)/propidium iodide (PI) apoptosis detection kit (Sangon Biotech, Shanghai, China) was utilized for detecting OA chondrocyte apoptosis. Following transfection for 48 h, OA chondrocytes were collected, washed, re-suspended, and stained with Annexin V-FITC (10 μL) and PI (5 μL). After incubation for 15 min in the dark, OA chondrocytes were then subjected to flow cytometry (Partec AG, Arlesheim, Switzerland) for measuring the rate of apoptotic cells.

### Western blot assay

RIPA lysis buffer (Beyotime) was utilized for extracting total protein. After quantification by BCA protein assay kit (Beyotime), protein lysates (about 40 μg) were loaded onto sodium dodecyl sulfate-polyacrylamide gel electrophoresis (SDS-PAGE) before being transferred to polyvinylidene difluoride membranes. These membranes were incubated with 5% non-fat milk to block non-specific binding. After that, the membranes were probed with specific primary antibodies at 4 °C for 12–16 h, and continuously probed with secondary antibody for 2 h. The antibodies including B cell lymphoma-2 (Bcl-2; 1:1000, ab196495), BCL2-associated X protein (Bax; 1:1000, ab77566), tumor necrosis factor alpha (TNF-α; 1:1000, ab9739), interleukin-6 (IL-6; 1:1000, ab208113), interleukin-1 beta (IL-1β; 1:1000, ab2105), MMP3 (1:1000, ab53015), collagen type II (1:2000, ab34712), GAPDH (1:2000, ab37168), and HRP-conjugated IgG anti-rabbit (1:4000, ab205718) were purchased from Abcam (Cambridge, UK); and caspase 3 (1:1000, #9662), poly (ADP-ribose) polymerase (PARP; 1:1000, #9532), and NAMPT (1:1000, #61122) were purchased from Cell Signaling Technology (Danvers, MA, USA). At last, the immune complexes were detected using the enhanced chemiluminescence reagent (Tanon, Shanghai, China). The protein levels were normalized by GAPDH, and ImageJ software was employed to assess the bands density.

### Dual-luciferase reporter assay

The potential complementary sequence of miR-127-5p and circ_0128846 or NAMPT was predicted by CircInteractome or Targetscan. Partial sequences of circ_0128846 or NAMPT 3’UTR containing wide-type (wt) or mutant (mut) miR-127-5p binding sites were synthesized and then cloned into the pmirGLO Dual-luciferase vectors (GenePharma, Shanghai, China), namely circ_0128846-wt, circ_0128846-mut, NAMPT-wt, and NAMPT-mut. OA chondrocytes were co-transfected with the constructed luciferase vector (wt or mut) and NC or miR-127-5p for 48 h. At last, luciferase activity was analyzed by dual-luciferase reporter assay system (Promega, Madison, WI, USA), followed by normalization to the Renilla luciferase.

### RNA immunoprecipitation (RIP) assay

To validate the interaction between miR-127-5p and circ_0128846 or NAMPT, RIP experiment was performed using the EZ-Magna RIP Kit (Millipore). In brief, OA chondrocytes were lysed by complete RIP lysis buffer. Afterwards, 100 μL of OA chondrocyte lysate was incubated by RIP buffer containing magnetic beads conjugated with human anti-Argonaute2 (Anti-Ago2) or anti-immunoglobulin G (Anti-IgG). After that, Proteinase K was applied to separate the immunoprecipitated RNAs. At last, qRT-PCR was performed for detecting the levels of miR-127-5p, circ_0128846, and NAMPT.

### Statistical analysis

All the experimental data from at least three independent experiments were displayed as mean ± standard deviation (SD). Statistical analyses were performed with GraphPad Prism 6.0. Student’s *t* test was used for evaluating the significance of differences between two groups or a one-way analysis of variance (ANOVA) was utilized to analyze significant differences among more than two groups. Pearson’s correlation coefficient analysis was employed to analyze the correlations among miR-127-5p, circ_0128846, and NAMPT. Statistical significance was considered when *P* < 0.05.

## Results

### Circ_0128846 was upregulated in OA cartilage tissues

**Fig. 1:**
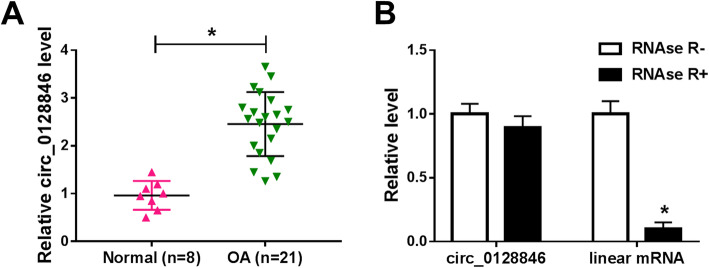
Circ_0128846 expression was increased in OA cartilage tissues. **a** The expression of circ_0128846 was determined by qRT-PCR analysis in OA cartilage tissues and normal cartilage tissues. **b** The levels of circ_0128846 and linear mRNA (ZFR) were determined after treatment of RNase R by qRT-PCR in OA chondrocytes. *P < 0.05

Firstly, we selected 5 upregulated circRNAs and tested their expression in normal and OA cartilage tissues. The expression of circ_0128846 was found to be the most upregulated (Supplementary Figure 1A), and its role in OA has not been reported. Therefore, circ_0128846 was selected for subsequent research. To explore the potential roles of circ_0128846 in OA, its expression was detected by qRT-PCR in OA cartilage tissues and normal cartilage tissues. The results showed that the expression of circ_0128846 was greatly increased in OA cartilage tissues compared to normal cartilage tissues (Fig. 1a). In general, RNase R can digest liner RNA but not circRNA. As displayed in Fig. 1b, linear mRNA (ZFR) was obviously decreased after digestion by RNase R and circ_0128846 expression was not affected, indicating the cyclic structure of circ_0128846.

### Knockdown of circ_0128846 increased cell viability and inhibited apoptosis, inflammation, and ECM degradation in OA chondrocytes

**Fig. 2:**
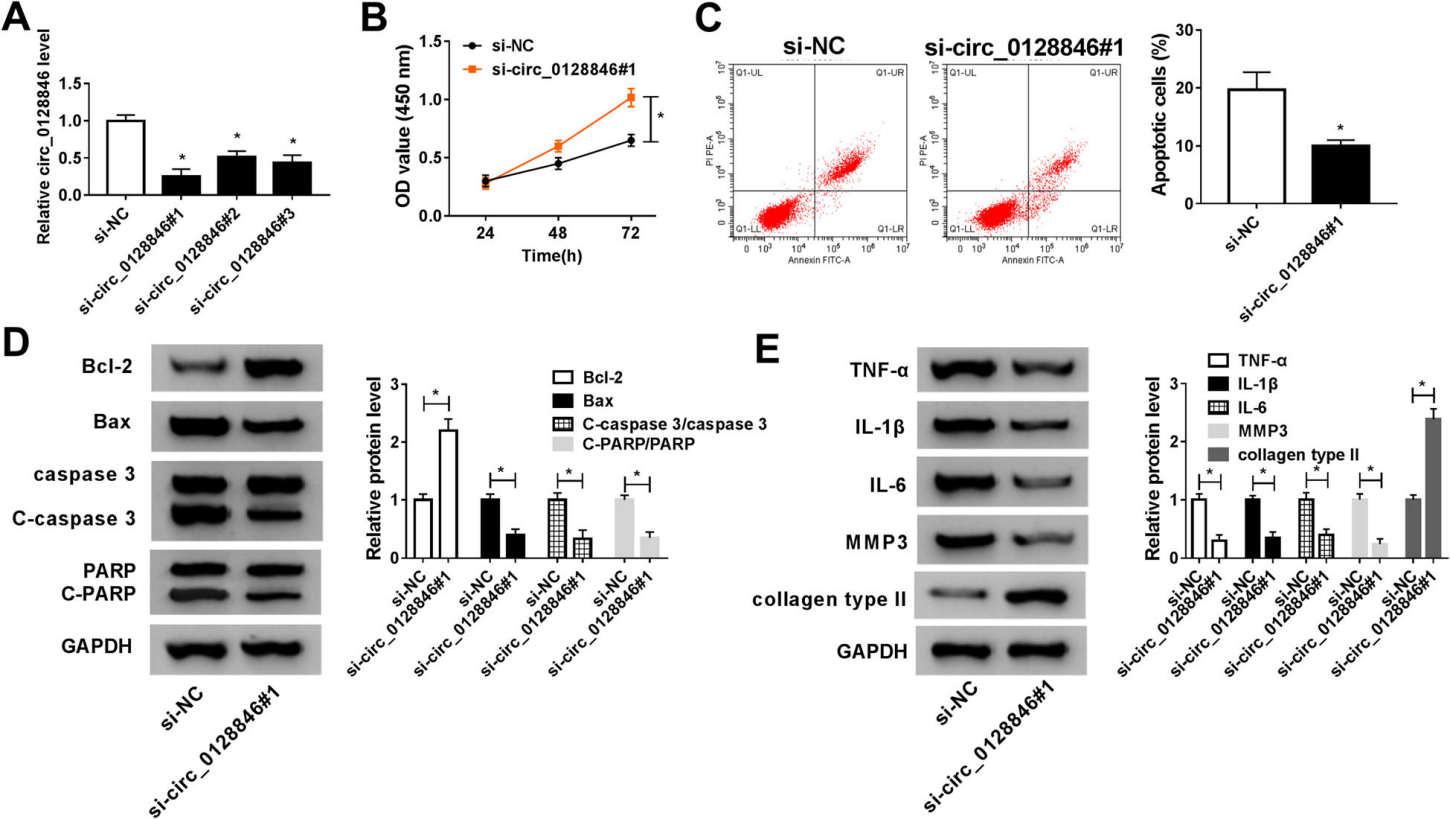
Circ_0128846 silence increased cell viability and suppressed apoptosis, inflammation, and ECM degradation in OA chondrocytes. **a** Knockdown efficiency of circ_0128846 was determined by qRT-PCR in OA chondrocytes transfected with si-NC, si-circ_0128846#1, si-circ_0128846#2, and si-circ_0128846#3. **b**–**e** OA chondrocytes were transfected with si-NC or circ_0128846#1. **b** Cell viability was assessed by CCK-8 assay. **c** Cell apoptosis was examined using flow cytometry analysis. **d** Western blot assay was conducted to measure the protein levels of Bcl-2, Bax, caspase 3, C-caspase 3, PARP, and C-PARP. **e** The protein levels of TNF-α, IL-1β, IL-6, MMP3, and collagen type II were detected by western blot analysis. *P < 0.05

To explore the effect of circ_0128846 on OA progression, functional experiments were performed in OA chondrocytes transfected with siRNAs. Knockdown efficiency of circ_0128846 was determined by qRT-PCR. As shown in Fig. 2a, compared with si-NC group, the expression of circ_0128846 was obviously reduced in OA chondrocytes transfected with si-circ_0128846#1, si-circ_0128846#2, or si-circ_0128846#3, especially in si-circ_0128846#1 group. Next, we chose si-circ_0128846#1 for further study. CCK-8 assay indicated that knockdown of circ_0128846 enhanced cell viability in OA chondrocytes (Fig. 2b). Moreover, cell apoptosis was reduced after downregulating circ_0128846 (Fig. 2c). Besides, the relative expression levels of apoptosis-related proteins, including Bcl-2 (anti-apoptotic molecule), Bax (pro-apoptotic molecule), cleaved-caspase 3 (C-caspase 3; a key executor in apoptotic process), and cleaved-PARP (C-PARP; pro-apoptotic protein) were analyzed by western blot assay. As presented in Fig. 2d, interference of circ_0128846 increased the protein level of Bcl-2, and decreased the protein expression of Bax, C-caspase 3/caspase 3 ratio, and C-PARP/PARP ratio. Besides, pro-inflammatory cytokines (TNF-α, IL-1β and IL-6), and ECM product (collagen type II), and catabolic enzyme (MMP3) were detected by western blot assay. The results showed that knockdown of circ_0128846 decreased the protein levels of TNF-α, IL-1β, IL-6, and MMP3 while increased the protein expression of collagen type II (Fig. 2e). These results indicated that circ_0128846 played a significant role in regulating cell viability, apoptosis, inflammatory response, and ECM degradation of OA chondrocytes.

### Circ_0128846 acted as a sponge of miR-127-5p

**Fig. 3:**
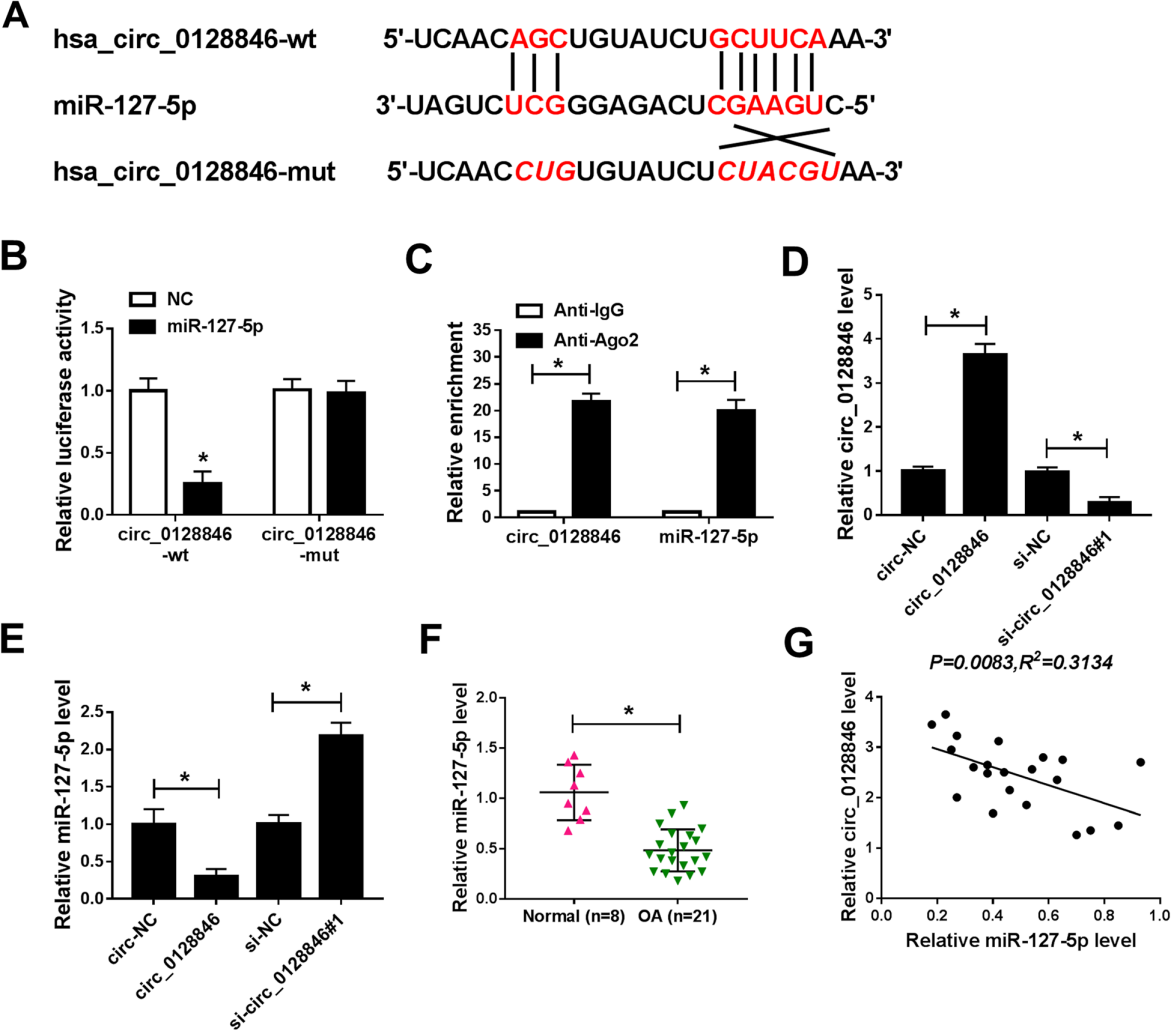
Circ_0128846 directly interacted with miR-127-5p. **a** The putative binding sites between circ_0128846 and miR-127-5p were predicted by CircInteractome. **b** Dual-luciferase luciferase reporter assay was used to detect the luciferase activity in OA chondrocytes co-transfected with circ_0128846-wt or circ_0128846-mut and NC or miR-127-5p. **c** The enrichment of circ_0128846 or miR-127-5p was measured by RIP assay in OA chondrocytes incubated with Anti-Ago2 or Anti-IgG. **d**, **e** The expression levels of circ_0128846 and miR-127-5p were examined by qRT-PCR in OA chondrocytes transfected with circ-NC, circ_0128846, si-NC, or si-circ_0128846#1. **f** The expression of miR-127-5p was analyzed in OA cartilage tissues and normal cartilage tissues by qRT-PCR. **g** The correlation between circ_0128846 and miR-127-5p expression was analyzed in OA cartilage tissues. *P < 0.05

Previous studies suggested that circRNAs could serve as sponges for miRNAs [25]. To determine whether circ_0128846 could serve as a sponge for miRNA, the potential targets of circ_0128846 were predicted by CircInteractome. There are many miRNAs targeted by circ_0128846. We selected five common miRNAs involved in the development of OA. We found that overexpression of circ_0128846 significantly downregulated the expression of miR-127-5p (Supplementary Figure 1B). Therefore, miR-127-5p was selected as the target of circ_0128846 for subsequent research. The putative binding sites miR-127-5p and circ_0128846 were shown in Fig. 3a. Dual-luciferase reporter and RIP assays were conducted to confirm this prediction. The results showed that miR-127-5p overexpression significantly reduced the luciferase activity of circ_0128846-wt but not circ_0128846-mut (Fig. 3b). Moreover, the results of RIP showed that the enrichment of circ_0128846 and miR-127-5p was obviously enhanced in Anti-Ago2 group compared to Anti-IgG group (Fig. 3c). The results of qRT-PCR indicated that transfection of circ_0128846 markedly increased the expression of circ_0128846, while transfection of si-circ_0128846#1 showed an opposite effect (Fig. 3d), suggesting that circ_0128846 and si-circ_0128846#1 were successfully transfected into OA chondrocytes. In addition, the expression of miR-127-5p was decreased after overexpression of circ_0128846, and knockdown of circ_0128846 promoted the expression of miR-127-5p (Fig. 3e). Furthermore, we observed that the level of miR-127-5p was reduced in OA cartilage tissues compared with normal cartilage tissues (Fig. 3f). In addition, correlation between miR-127-5p and circ_0128846 expression was analyzed in OA cartilage tissues. As displayed in Fig. 3g, a negative correlation between miR-127-5p and circ_0128846 expression was observed in OA cartilage tissues (*P* = 0.0083, *R*^*2*^ = 0.3134). All these data indicated that miR-127-5p was a direct target of circ_0128846.

### MiR-127-5p knockdown reversed the effects of si-circ_0128846#1 on cell viability, apoptosis, inflammation, and ECM degradation in OA chondrocytes

**Fig. 4:**
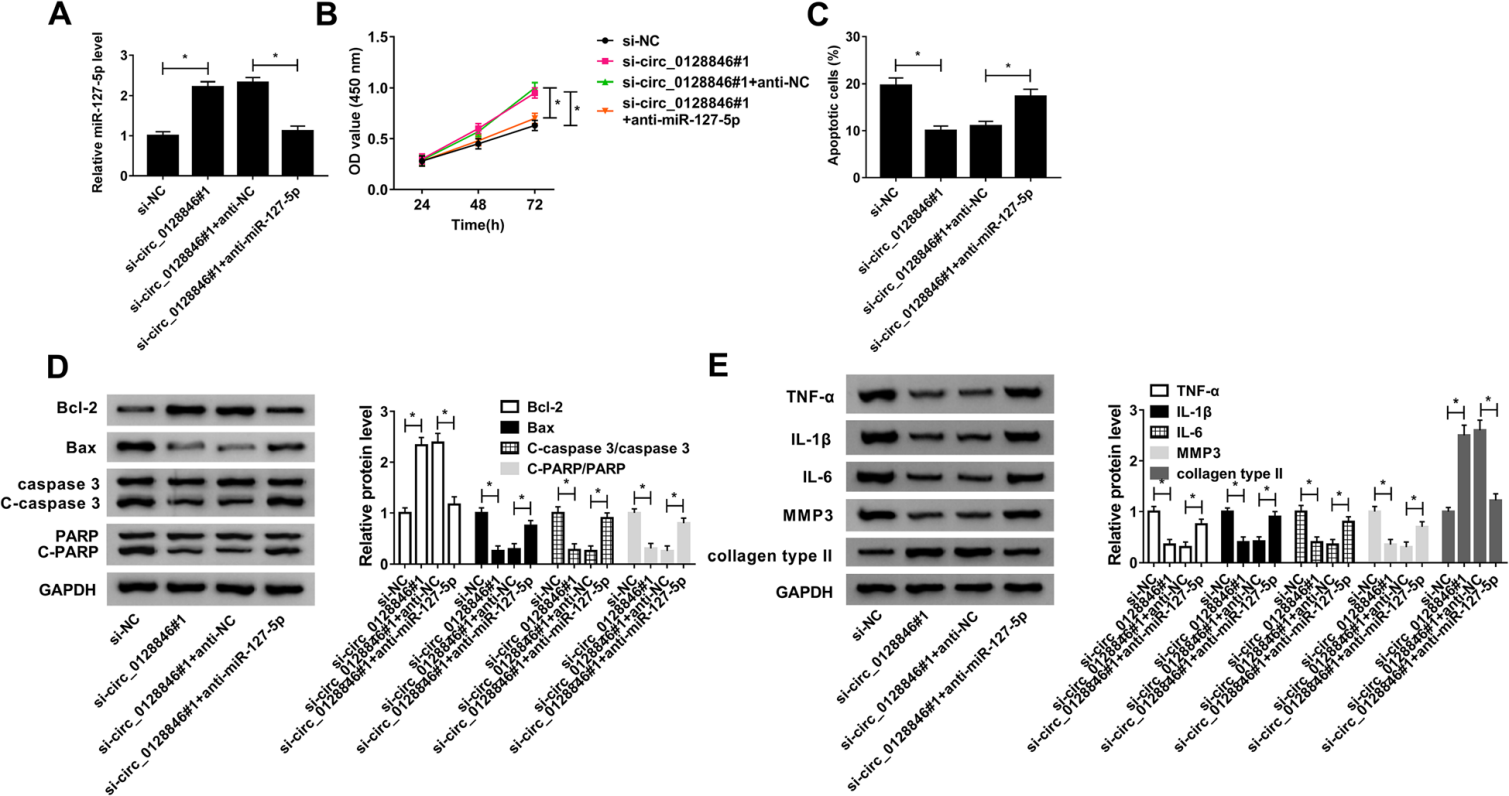
Circ_0128846 exerted its functions by sponging miR-127-5p in OA chondrocytes. OA chondrocytes were transfected with si-NC, si-circ_0128846#1, si-circ_0128846#1 + anti-NC, or si-circ_0128846#1 + anti-miR-127-5p. **a** The level of miR-127-5p was examined by qRT-PCR. **b** CCK-8 assay was conducted to evaluate cell viability. **c** Flow cytometry analysis was employed to measure the apoptosis rate. **d** Protein levels of Bcl-2, Bax, caspase 3, C-caspase 3, PARP, and C-PARP were examined by western blot assay. **e** The protein levels of TNF-α, IL-1β, IL-6, MMP3, and collagen type II were determined using western blot analysis. *P < 0.05

To explore whether the biological effects of circ_0128846 were mediated by miR-127-5p, rescue experiments were performed. Knockdown of circ_0128846 promoted the expression of miR-127-5p, which was reversed by downregulating miR-127-5p (Fig. 4a). CCK-8 assay indicated that the promoting effect of circ_0128846 downregulation on cell viability was abolished by knockdown of miR-127-5p (Fig. 4b). Moreover, the inhibitory effect of circ_0128846 silence on apoptosis was rescued by downregulation of miR-127-5p (Fig. 4c). Furthermore, miR-127-5p interference abated the effect of circ_0128846 knockdown on promoting Bcl-2 expression and reducing Bax expression, C-caspase 3/caspase 3 ratio, and C-PARP/PARP ratio (Fig. 4d). In addition, the reduction of TNF-α, IL-1β, IL-6, and MMP3 expression and promotion of collagen type II expression caused by transfection with si-circ_0128846#1 were reversed by co-transfection with anti-miR-127-5p (Fig. 4e). Taken together, these data suggested that circ_0128846 exerted its biological roles in OA chondrocytes by sponging miR-127-5p.

### NAMPT was a direct target gene of miR-127-5p

**Fig. 5:**
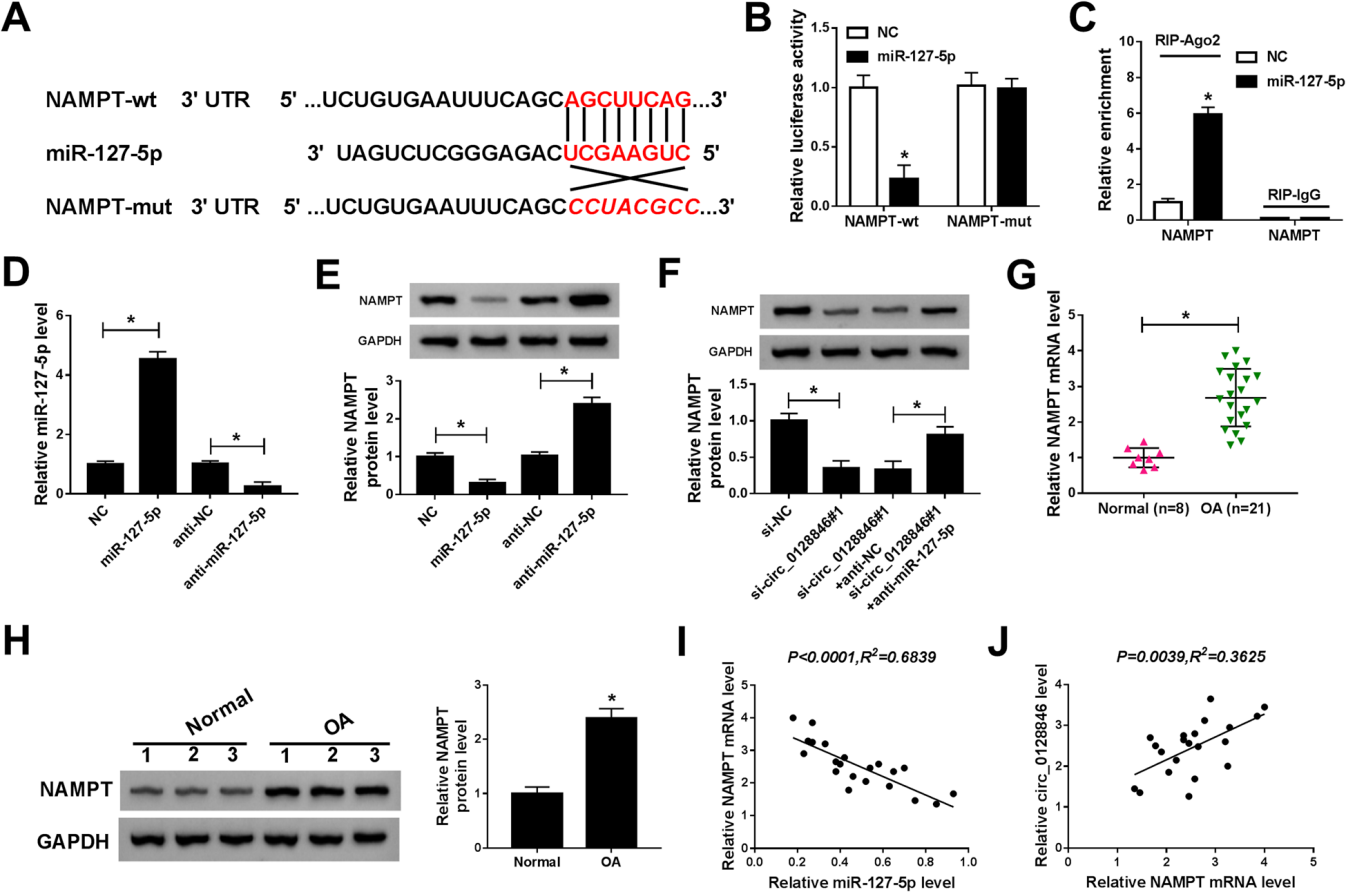
NAMPT was a downstream target of miR-127-5p. **a** The predicted binding sites and its mutant for miR-127-5p on the 3′UTR of NAMPT were shown. **b** Relative luciferase activity was determined in OA chondrocytes co-transfected with NAMPT-wt or NAMPT-mut and NC or miR-127-5p. **c** The enrichment of NAMPT was measured by RIP assay in OA chondrocytes transfected with NC or miR-127-5p. **d**, **e** The level of miR-127-5p and protein level of NAMPT were determined by qRT-PCR and western blot analyses, respectively, in OA chondrocytes transfected with NC, miR-127-5p, anti-NC, or anti-miR-127-5p. **f** The protein expression of NAMPT was examined by western blot assay in OA chondrocytes transfected with si-NC, si-circ_0128846#1, si-circ_0128846#1 + anti-NC, or si-circ_0128846#1 + anti-miR-127-5p. **g**, **h** NAMPT mRNA and protein levels were measured in OA cartilage tissues and normal cartilage tissues by qRT-PCR and western blot analyses, respectively. **i** The association between NAMPT mRNA level and miR-127-5p abundance was analyzed in OA cartilage tissues. **j** The correlation between circ_0128846 level and NAMPT mRNA level was analyzed in OA cartilage tissues. *P < 0.05

MiRNAs control many biological processes through direct interaction with their target mRNAs [26]. Hence, Targetscan online website was utilized to search for the potential target mRNAs of miR-127-5p. The prediction results showed that 3′UTR of NAMPT shared binding sites for miR-127-5p (Fig. 5a), suggesting NAMPT could possibly interact with miR-127-5p. To validate this assumption, dual-luciferase reporter and RIP assays were performed. The results indicated that introduction of miR-127-5p markedly decreased the luciferase activity of NAMPT-wt, whereas no change was observed in the luciferase activity of NAMPT-mut (Fig. 5b). Meanwhile, NAMPT was more abundant in chondrocytes  transfected with miR-127-5p in Ago2 pellet, indicating the interaction between NAMPT and miR-127-5p (Fig. 5c). The results of qRT-PCR showed that the expression of miR-127-5p was strikingly increased in OA chondrocytes transfected with miR-127-5p, while transfection of anti-miR-127-5p presented an opposite effect (Fig. 5d), indicating that transfection of miR-127-5p and anti-miR-127-5p was successful. Next, we explored the effect of miR-127-5p on NAMPT expression. Western blot assay revealed that overexpression of miR-127-5p inhibited the protein expression of NAMPT, and knockdown of miR-127-5p promoted the protein expression of NAMPT (Fig. 5e). Moreover, silence of circ_0128846 reduced the protein level of NAMPT, which could be reversed by downregulating miR-127-5p (Fig. 5f), suggesting that circ_0128846 regulated the expression of NAMPT by sponging miR-127-5p. Next, we investigated the expression of NAMPT in OA and normal cartilage tissues. The results showed that NAMPT mRNA level and protein level were increased in OA cartilage tissues relative to normal cartilage tissues (Fig. 5g, h). Furthermore, we analyzed the correlation between NAMPT mRNA level and miR-127-5p or circ_0128846 expression in OA cartilage tissues. As presented in Fig. 5i, j, NAMPT mRNA level was negatively correlated with miR-127-5p expression (*P* < 0.0001, *R*^*2*^ = 0.6839) and positively correlated with circ_0128846 level (*P* = 0.0039, *R*^*2*^ = 0.3625). These data collectively demonstrated that NAMPT could directly bind to miR-127-5p.

### MiR-127-5p overexpression increased cell viability and suppressed apoptosis, inflammation, and ECM degradation by downregulating NAMPT in OA chondrocytes

**Fig. 6:**
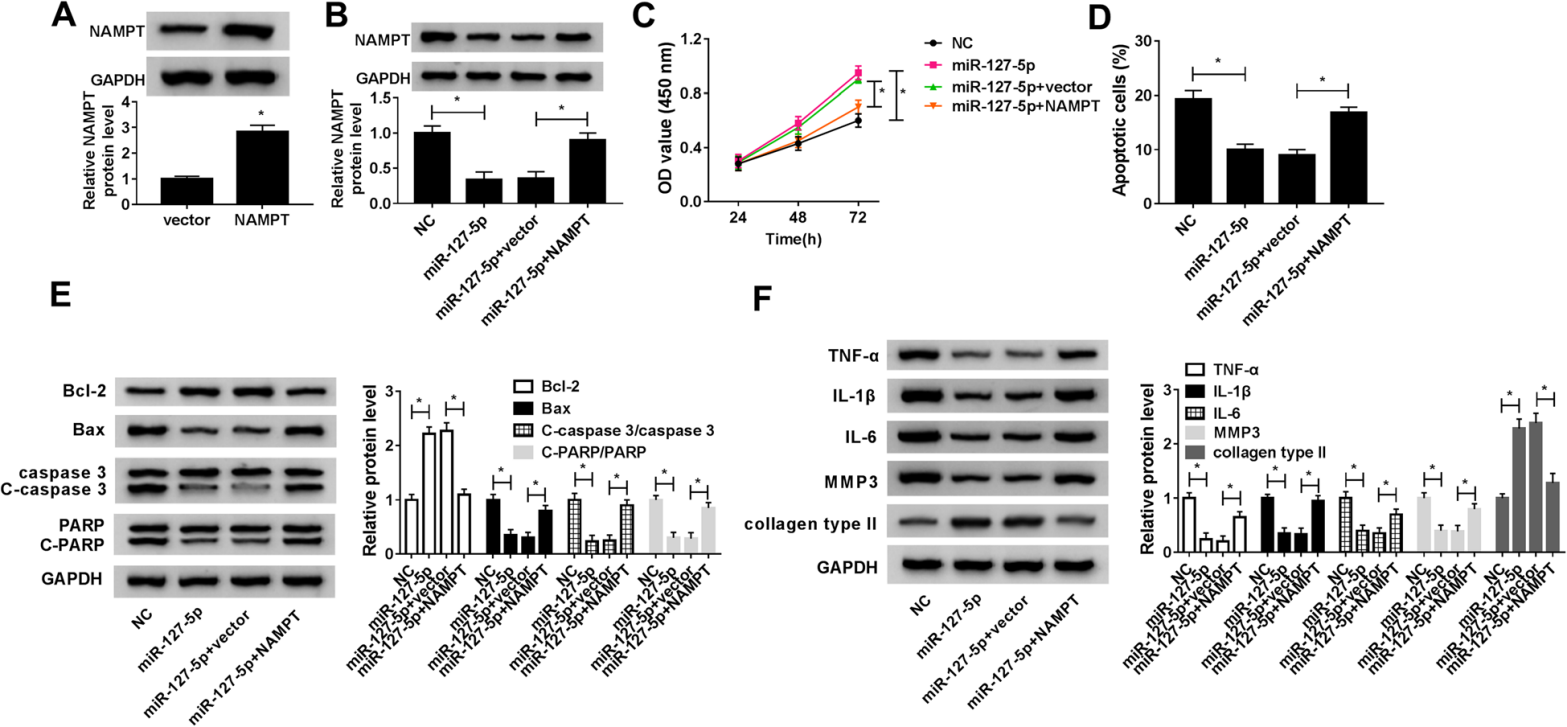
MiR-127-5p exerted its functions in OA chondrocytes by targeting NAMPT. **a** Overexpression efficiency of NAMPT was determined by western blot assay in OA chondrocytes transfected with NAMPT or vector. **b**–**f** OA chondrocytes were transfected with NC, miR-127-5p, miR-127-5p + vector, or miR-127-5p + NAMPT. **b** Western blot assay was performed to detect the protein expression of NAMPT. **c** Cell viability was evaluated by CCK-8 assay. **d** Cell apoptosis was determined using flow cytometry analysis. **e** The protein levels of Bcl-2, Bax, caspase 3, C-caspase 3, PARP, and C-PARP were analyzed by western blot assay. **f** Western blot assay was carried out to examine the protein expression of TNF-α, IL-1β, IL-6, MMP3, and collagen type II. *P < 0.05

Western blot assay was used to detect the transfection efficiency of NAMPT. The data showed that NAMPT was successfully overexpressed after transfection with NAMPT (Fig. 6a). To explore whether miR-127-5p exerted its biological functions by targeting NAMPT, OA chondrocytes were transfected with NC, miR-127-5p, miR-127-5p + vector, or miR-127-5p + NAMPT. Overexpression of miR-127-5p inhibited the protein expression of NAMPT, which was restored by addition of NAMPT (Fig. 6b). Moreover, overexpression of miR-127-5p increased cell viability and inhibited cell apoptosis, which could be reversed by upregulation of NAMPT (Fig. 6c, d). In addition, miR-127-5p restoration increased the protein level of Bcl-2 and decreased the protein expression of Bax expression, C-caspase 3/caspase 3 ratio, and C-PARP/PARP ratio, whereas these effects were abated by upregulating NAMPT (Fig. 6e). Furthermore, the protein levels of TNF-α, IL-1β, IL-6, and MMP3 were reduced and collagen type II expression was increased after transfection with miR-127-5p, while co-transfection with NAMPT abolished these effects (Fig. 6f). Altogether, these data proved that miR-127-5p exerted its biological roles in OA chondrocytes by targeting NAMPT.

## Discussion

OA is the most prevalent age-related joint disorder, and it brings a huge life and economic burden to OA patients [27]. The secretion of inflammatory factors has been demonstrated to play crucial roles in the pathological process of OA [28]. Besides, chondrocyte apoptosis and ECM degradation are closely related to OA progression [29]. In this report, we aimed to study the biological roles and regulatory mechanism of circ_0128846, miR-127-5p, and NAMPT in OA progression.

As a kind of newly discovered non-coding RNAs, circRNAs are more stable and difficult to degrade due to the covalently closed loop structures [30]. CircRNAs are recognized as important therapeutic targets and new biomarkers in many diseases [31]. The abnormal expression of circRNAs was closely associated with occurrence and development of OA [32]. For instance, Zhou et al. found that circRNA.33186 promoted OA pathogenesis via sponging miR-127-5p [33]. Li et al. revealed that hsa_circ_0045714 regulated cell growth, apoptosis, and ECM synthesis in chondrocytes through regulating miR-193b/IGF1R axis [34]. A previous study suggested that circ_0128846 was upregulated in OA [12]. However, the effect of circ_0128846 on OA progression remains unclear. Here, we also found that circ_0128846 expression was enhanced in OA cartilage tissues. Functionally, interference of circ_0128846 increased cell viability and inhibited apoptosis, inflammation, and ECM degradation in OA chondrocytes, suggesting that inhibition of circ_0128846 might be a promising strategy for treatment of OA.

Accumulating evidence has suggested that circRNAs participate in the regulation of many diseases via acting as miRNA sponges [35]. In recent years, many miRNAs have been demonstrated to play critical roles in OA progression through modulating ECM anabolism and chondrocyte catabolism [36]. To confirm whether circ_0128846 served as miRNAs sponges in OA, bioinformatics software was employed to predict the possible target miRNAs of circ_0128846. The data showed that miR-127-5p was a possible target of circ_0128846, which was verified through performing dual-luciferase reporter and RIP assays. The former study demonstrated that miR-127-5p interference abolished the inhibitory effect of si-circRNA.33186 on OA progression [33]. Besides, Li et al. reported that miR-127-5p was downregulated in OA cartilage tissues, and miR-127-5p overexpression negatively regulated MMP13 expression to enhance OA chondrocyte proliferation [12]. In line with these findings, we also observed that miR-127-5p level was declined in OA cartilage samples, and inhibition of miR-127-5p abated the effects of circ_0128846 interference on cell viability, apoptosis, inflammation, and ECM degradation in OA chondrocytes. These results suggested that the effects of circ_0128846 downregulation in OA chondrocytes were mediated by miR-127-5p.

It is widely acknowledged that miRNAs exert their functions via suppressing the expression of target mRNAs [37]. So, the possible targets of miR-127-5p were predicted using TargetScan online website. Our results proved that NAMPT was a target of miR-127-5p. More and more reports have demonstrated that NAMPT has a catabolic function in cartilage and also play a crucial role in the progression of OA [38]. NAMPT has also been shown to be an important player in inflammatory arthritis [39]. More importantly, Wu et al. demonstrated that hsa_circ_0005105 increased the expression of NAMPT and promoted chondrocyte ECM degradation via sponging miR-26a [40]. These findings revealed that NAMPT has a vital role in OA progression. In this study, the data showed that NAMPT expression was elevated in OA cartilage tissues. The rescue experiments indicated that NAMPT overexpression could reverse the impact of miR-127-5p on promotion of cell viability and reduction of apoptosis, inflammation and ECM degradation in OA chondrocytes, suggesting miR-127-5p exerted its functions by targeting NAMPT. Mechanistically, circ_0128846 positively regulated NAMPT expression via sponging miR-127-5p. Collectively, these data indicated that circ_0128846 might promote OA progression by regulating miR-127-5p/NAMPT axis. However, the role of circ_0128846/miR-127-5p/NAMPT axis in OA is still required to be further confirmed in animal models in the future study. In addition, since no microarray analysis has been performed, we could not identify more dysregulated circRNAs and miRNAs in OA, and more circRNAs–miRNAs-mRNAs regulatory networks have not been discussed. In future work, we hope that microarray analysis can be carried out for further research in OA.

## Conclusion

In conclusion, our research indicated that circ_0128846 and NAMPT were overexpressed and miR-127-5p was lowly expressed in OA cartilage tissues. Moreover, circ_0128846 knockdown increased cell viability and suppressed apoptosis, inflammation and ECM degradation in OA chondrocytes by upregulating miR-127-5p and downregulating NAMPT. Our study is the first to elucidate the circ_0128846/miR-127-5p/NAMPT regulatory network in OA chondrocytes, leading to better understanding of OA progression and offering a possible target for gene therapy.

## Supplementary Information

**Additional file 1: Figure S1**. Relative expression of 5 circRNAs in OA cartilage tissues, and the effect of circ_0128846 on the expression of potential target miRNAs. (A) The expression levels of circ_0128846, circ_0114876, circ_0128006, circ_0136474, and circ_0001721 in normal and OA cartilage tissues were detected by qRT-PCR. (B) The expression levels of circ_0136474, miR-127-5p, miR-338-3p, miR-183-5p, miR-197, and miR-153-3p were measured by qRT-PCR in OA chondrocytes transfected with circ-NC or circ_0136474. *P<0.05.

## Data Availability

The analyzed data sets generated during the present study are available from the corresponding author on reasonable request.
