# Supplementary figures and images for: Circular RNA circ_0128846 promotes the progression of osteoarthritis by regulating miR-127-5p/NAMPT axis
Source: J Orthop Surg Res. 2021 May 11;16:307. doi: 10.1186/s13018-021-02428-z (PMC8112058; doi:10.1186/s13018-021-02428-z)

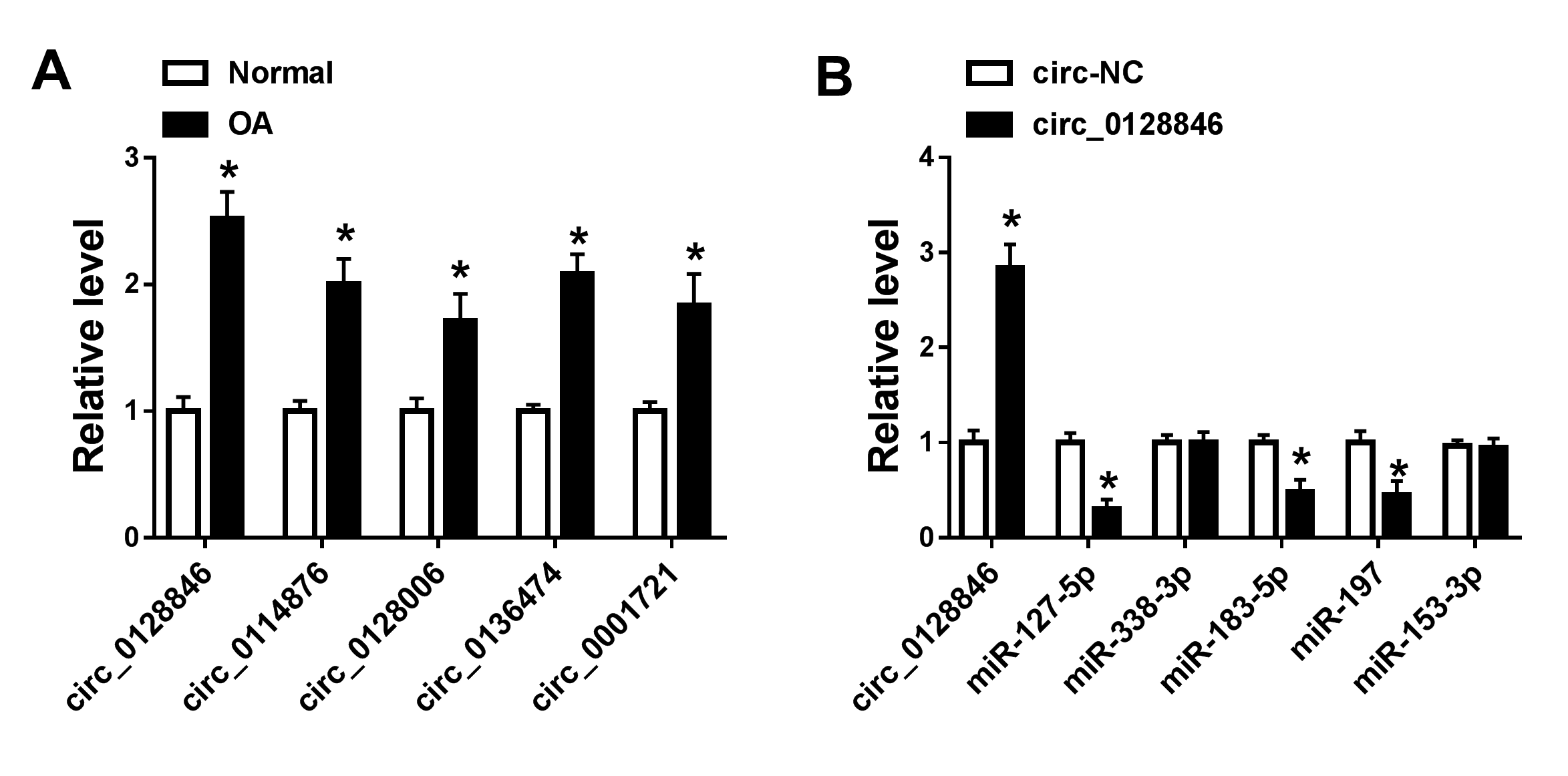

Supplement: Supplementary file 1 — Additional file 1: Figure S1. Relative expression of 5 circRNAs in OA cartilage tissues, and the effect of circ_0128846 on the expression of potential target miRNAs. (A) The expression levels of circ_0128846, circ_0114876, circ_0128006, circ_0136474, and circ_0001721 in normal and OA cartilage tissues were detected by qRT-PCR. (B) The expression levels of circ_0136474, miR-127-5p, miR-338-3p, miR-183-5p, miR-197, and miR-153-3p were measured by qRT-PCR in OA chondrocytes transfected with circ-NC or circ_0136474. *P<0.05. [file 13018_2021_2428_MOESM1_ESM.tif]
